# Supplementary material for: Vacancy driven surface disorder catalyzes anisotropic evaporation of ZnO (0001) polar surface
Source: Nat Commun. 2022 Sep 24;13:5616. doi: 10.1038/s41467-022-33353-2 (PMC9509323; doi:10.1038/s41467-022-33353-2)
Supplement: Supplementary file 2 — Description of Additional Supplementary Files [file 41467_2022_33353_MOESM2_ESM.docx]

**Description of Additional Supplementary Files**

File Name: Supplementary Movie 1

Description: Low temperature evaporation of ZnO as observed by HRTEM at 150 °C

File Name: Supplementary Movie 2

Description: Anisotropic evaporation of ZnO observed by in-situ HRTEM at 300 °C

File Name: Supplementary Movie 3

Description: Video - NCSI HRTEM movie showing anisotropic evaporation of ZnO at 300 °C

File Name: Supplementary Movie 4

Description:  O-terminated and non-polar ZnO evaporation at 300 °C

File Name: Supplementary Movie 5

Description: NCSI HRTEM movie showing the atomistic details of disordering of (0001) surface at 300 °C

File Name: Supplementary Movie 6

Description: NCSI HRTEM movie showing the displacement of Zn and O atomic columns triggered by diffusion of Zn vacancies before the loss of long-range order.

File Name: Supplementary Movie 7

Description:  Collective motion from atomic displacement of Zn and O columns in Zn-deficient subsurface

File Name: Supplementary Movie 8

Description:  Real-time strain mapping of NCSI HRTEM movie of ZnO during evaporation

File Name: Supplementary Movie 9

Description:  Ab initio MD simulations showing VZn induced lattice distortion and disordered quasi-liquid layer formation
