# Supplementary material for: Vacancy driven surface disorder catalyzes anisotropic evaporation of ZnO (0001) polar surface
Source: Nat Commun. 2022 Sep 24;13:5616. doi: 10.1038/s41467-022-33353-2 (PMC9509323; doi:10.1038/s41467-022-33353-2)
Supplement: Supplementary file 3 — Supplementary Information [file 41467_2022_33353_MOESM3_ESM.pdf]

Supplementary Information

Vacancy driven surface disorder catalyzes anisotropic evaporation of ZnO (0001) polar surface

Zhen Wang†, Jinho Byun†, Subin Lee, Jinsol Seo, Bumsu Park, Jong Chan Kim, Hu Young Jeong, Junhyeok Bang*, Jaekwang Lee*, Sang Ho Oh*

*Correspondence and requests for materials should be addressed to:

J.B. (email: jbang@cbnu.ac.kr); J.L. (email: jaekwangl@pusan.ac.kr); S.H.O. (email: shoh@kentech.ac.kr)

†These authors contributed equally to this work.

Supplementary Note1 to 6

Supplementary Table 1, 2

Supplementary Figure 1 to 21

Supplementary Movie legends

Supplementary References

Supplementary Note 1. HRTEM simulation and quantitative analysis.

Many factors influence the contrast of ZnO surface profile HRTEM images. To determine the exact HRTEM imaging parameters, including specimen thickness, specimen tilt, absorption, and optical aberrations, iterative HRTEM simulation has been utilized by comparing with experimental HRTEM images[1]. A redefined orthogonal ZnO unit-cell along $[11\bar{2}0]$ direction (Supplementary Fig. S2a) was built for the multi-slice HRTEM image simulation. A thickness-defocus map shown in Supplementary Fig. S2b was compared with the experimental images to determine the defocus and thickness.

The defocus ranges from 0 to +9 nm and the thickness range from 1 to 20 unit cells represent the typical NCSI HRTEM image contrast of ZnO observed in our experiments. When the Zn and O column intensity are plotted as a function of thickness (number of atoms in the column) at the defocus of +5.2 nm, a linear relationship of the intensity variation is valid up to 10 Zn atoms and 25 O atoms (Supplementary Fig. S2c). To determine the exact number of Zn and O atoms at the defocus of +5.2 nm, an interactive imaging matching between experimental and simulated images has been carried out[2]. The best matching condition yielding the maximum cross-correlation coefficient (XCC, 0.975) between experimental and simulated images was determined to be 5 unit cells (Supplementary Fig. S2d). For the image simulation the modulation transfer function (MTF) of CCD camera (OneView, Gatan) has been taken into account[3,4]. Comparison of the intensity profiles of both Zn and O atoms in experimental and simulation images yield a good matching (Supplementary Fig. S2e).

Supplementary Note 2. Contamination of the ZnO sample at in-situ heating conditions in TEM

As shown in the EELS and EDS spectra (Supplementary Figs. S7a, d), no elements other than Zn and O were not detected. The carbon concentration is estimated to be less than 0.6 % by EDS quantification, which is in the range of detection limit of EDS[5] (Table S1).

Supplementary Table 1. Quantification of the EDS data obtained from ZnO bulk region. While the concentration (atomic %) of O and Zn is close to 50% and remain stoichiometric, the concentration of carbon (C) is negligibly small (< 0.6%).

Element	Energy (keV)	Counts	Mass%	Atom%
C	K_α, 0.277	9.81	0.17	0.55
O	K_α, 0.525	1823.56	20.29	50.76
Zn	K_α, 8.63	4397.17	79.55	48.69
Total	-	-	100	100

Supplementary Note 3. Electron beam induced displacement rate of Zn and O atoms

The high energy (300 keV) electron can displace surface Zn and O atoms and thereby influence the dissociative evaporation behavior of ZnO. We calculated the electron beam induced displacement rates of Zn and O atoms to evaluate their effects on the experimentally observed evaporation rate. We considered the two electron beam damage mechanisms for Zn and O atoms in ZnO[6], which are: (1) knock-on displacement and (2) radiolytic displacement. Following the method described by Lawrence et al.[7], we calculated the elastic and the inelastic scattering cross sections (σ) for knock-on and radiolytic displacement of Zn and O atoms, respectively. To estimate the rate of Zn and O atoms that are displaced by the electron beam, the scattering cross section was multiplied by the electron dose rate. The procedure is briefly described for knock-on and radiolytic displacement as follows.

(1) Knock-on displacement rate

The scattering between high energy electron and atomic nucleus is mostly elastic. The initially rest surface atoms can be displaced when the transferred energy (E) is larger than the desorption energy. The transferred energy of electron to a target atom shows a strong angular dependency according to the following relationship:

$$E = E_{\max} \sin^2(\theta/2)$$
$$E_{\max} = 2E_0\left(E_0 + 2m_0c^2\right)/Mc^2$$

where E_{max} is the maximum possible energy transfer, corresponding to head-on collision, i.e., scattering angle θ = 180°, M is the mass of the target atom, and E_0 is the kinetic energy of the incident electron (300 keV), c is the speed of light and m_0c^2 = 511 keV is the electron rest energy. For Zn and O atoms E_{max} is calculated to be 13.0 eV and 53.1 eV, respectively. For Zn and O atoms to be ejected from the surface the minimum energy transfer (E_{min}) must be greater than or equal to the desorption energy (E_a). E_a is chosen as a variable to evaluate how the displacement rate depends on the desorption energy of Zn and O atoms with different surface orientations.

The differential scattering cross section ($d\sigma/d\theta$) derived for Rutherford scattering model which ignores the screening effect of nucleus field by surrounding electrons has been used.

$$\frac{d\sigma}{d\theta} = \left(\frac{e^2}{8\pi\varepsilon_0 E_0}\right)^2 \left(\frac{E_0 + mc_0^2}{E_0 + 2mc_0^2}\right)^2 \left[\frac{2\pi \sin\theta}{\sin^4(\theta/2)}\right]$$

This expression can be integrated over the scattering angle, from θ = 180° to a minimum value given by $\sin^2(\theta/2) = E_{min}/E_{max}$, to give a cross section for energy transfer in the range E_{min} to E_{max}.

Nuclear field, which is a good approximation for large scattering angles, the differential scattering cross section for such -type scattering is: give a cross section for energy transfer in the range E_{min} to E_{max}:

$$\sigma = \left(2.45\times10^{-29}\text{m}^2\right)Z^2\left[\frac{1-v^2/c^2}{\left(v^2/c^2\right)^2}\right]\times\left[\left(E_{\max}/E_{\min}\right)-1\right]$$

Elastic scattering cross section for Zn:

$$\sigma = \left(2.3933\times10^{-6}\,Å^2\right)\left[\left(13\text{eV}/E_a\right)-1\right]$$

Elastic scattering cross section for O:

$$\sigma = \left(1.7017\times10^{-7}\,Å^2\right)\left[\left(53.1\text{eV}/E_a\right)-1\right]$$

For an electron dose rate of D (e^- $Å^{-2}$ s^{-1}) and the elastic cross section σ, the number of damage events per unit volume (or surface area in the case of sputtering), N_k, is given by $N_k = D\,\sigma\,N_0$, where N_0 is the number of target atoms per unit volume (or surface area in the case of sputtering). A more general quantity that can be used for critical flux calculations is the number (or fraction) of displacement events per target atom, $x = N_k / N_0 = D\,\sigma$. In our experiments, a flux of 2×10^4 e^- $Å^{-2}$ s^{-1} was used, which gives the following formula for knock-on displacement rate.

For zinc: $D\sigma = \left(2.2\times10^4\,\frac{e^-}{Å^2 s}\right)\times\left(2.3933\times10^{-6}\right)\left[\left(13\text{ eV}/E_a\right)-1\right]$

For oxygen: $D\sigma = \left(2.2\times10^4\,\frac{e^-}{Å^2 s}\right)\times\left(1.7017\times10^{-7}\right)\left[\left(53.1\text{ eV}/E_a\right)-1\right]$

Those are shown by olive and orange colored solid line in Supplementary Fig. S9b, respectively.

(2) Radiolytic displacement rate

Radiolytic displacement of Zn and O atoms are induced by inelastic scattering between the incident high energy electron and the electrons surrounding the nucleus. The inelastic displaceme nt cross section is:

$$\sigma = 7\times10^6\,\xi\left(\frac{Z}{E_a}\right)*\left(10^{-28}\,\frac{m^2}{barns}\right)$$

ξ is the efficiency factor and is typically an empirically derived value. Here we adopted the same estimated value for CeO_2 in Lawrence et al[7] which is 10^{-5}.

By taking account of electron dose rate, the radiolytic displacement rate are given as follows:

For zinc: $D\sigma = \left(2.2\times10^{4}\,\frac{\mathrm{e}^{-}}{\mathring{A}^{2}s}\right)\times\left(2.1\times10^{-5}\right)\left(\frac{1}{E_a}\right)\mathring{A}^{2}$

For oxygen: $D\sigma = \left(2.2\times10^{4}\,\frac{\mathrm{e}^{-}}{\mathring{A}^{2}s}\right)\times\left(5.6\times10^{-6}\right)\left(\frac{1}{E_a}\right)\mathring{A}^{2}$

Those are shown by red and purple colored solid lines Supplementary Fig. S9b, respectively.

The calculated electron beam displacement rates are compared with the thermally activated desorption rate of surface Zn atoms and diffusion rate of the resulting V_{Zn}. We assume that the desorption rate of surface Zn atoms follows an Arrhenius type equation such as,

$$R = A\exp\left(-\frac{E_a}{k_B T}\right)$$

where A is the attempt frequency, T is the temperature, k_B is the Boltzmann constant, and E_a is the activation energy. The attempt frequency can be approximated by the average atomic vibrational frequency of 10^{13} Hz[7]. This equation is plotted as a function of E_a for T = 25, 300, 500 and 600 °C in Supplementary Fig. S9b. The desorption energy of Zn atom on (0001)-Zn polar surface (0.3 eV) and the energy barrier for inward diffusion of the resulting V_{Zn} (0.7 eV) are indicated in Supplementary Fig. S9b.

Supplementary Note 4. Elastic constants calculation.

To understand the anisotropic, collective atomic displacements of the near-surface region of ZnO (0001) surface before the loss of long-range order, we theoretically studied the lattice softening by the accumulation of V_{Zn} by calculating the elastic constants. To incorporate V_{Zn}, we considered a supercell containing 96 host atoms for the wurtzite ZnO structure. The 2×2×2 Monkhorst-Pack k-point mesh was used for Brillouin-zone integration. The V_{Zn} were randomly distributed on the supercell, and three samples with different vacancy distribution are considered for each Zn deficient case (one example shown in Supplementary Fig. S15), except the pure ZnO, and the calculated elastic constants are averaged for the three samples. In terms of the symmetry of wurtzite ZnO structure, the elastic constants reflecting the stress-strain relation can be expressed in the matrix form:

$$\begin{bmatrix} \sigma_1 \\ \sigma_2 \\ \sigma_3 \\ \sigma_4 \\ \sigma_5 \\ \sigma_6 \end{bmatrix} = \begin{bmatrix} C_{11} & C_{12} & C_{13} & 0 & 0 & 0 \\ C_{12} & C_{11} & C_{13} & 0 & 0 & 0 \\ C_{13} & C_{13} & C_{33} & 0 & 0 & 0 \\ 0 & 0 & 0 & C_{44} & 0 & 0 \\ 0 & 0 & 0 & 0 & C_{44} & 0 \\ 0 & 0 & 0 & 0 & 0 & C_{66} \end{bmatrix} \begin{bmatrix} \varepsilon_1 \\ \varepsilon_2 \\ \varepsilon_3 \\ \varepsilon_4 \\ \varepsilon_5 \\ \varepsilon_6 \end{bmatrix}$$

where σ_i and ε_i (i = 1, …,6) represent the stress and strain, respectively. Here, wurtzite ZnO has only five independent elastic constants and the additional linearly dependent $C_{66} = (C_{11} - C_{12})/2$. As shown in Supplementary Fig. S16, the DFT results show that all elastic constants of $Zn_{1-x}O$ decrease almost linearly with the Zn deficiency x. As more V_{Zn} diffuse into bulk region, the lattice are softened in Zn deficient region. As we discussed in the main text and Fig. 4e, C_{66} decreases faster than C_{44}. The results explain the experimentally observed anisotropic collective motion.

Supplementary Note 5. Atomic models for the Zn-deficient quasi-liquid

From the EELS and EDS quantification, the Zn:O ratio of the quasi-liquid layer is close to 0.43:1, which is close to the ratio of ZnO_2 peroxide (Fig. 3c). The atomic structure of liquid is not well defined, and DFT calculations for desorption from such a liquid layer is improper. Instead, to mimic the quasi-liquid layer, the two crystal structures of ZnO_2, i.e., cubic and orthorhombic as shown in Supplementary Figs. S20a, b, was placed on the (0001) ZnO surface. For the cubic ZnO_2, the [110] direction was fitted with the ZnO $[11\bar{2}0]$ direction. For the orthorhombic ZnO_2, the [010] direction was fitted with the ZnO $[11\bar{2}0]$ direction. The cubic and orthorhombic layer thicknesses were 7.2 and 9.1 Å, respectively (Supplementary Figs. S20c, d).

Supplementary Note 6. Comparison of the LDA+U with HSE functional calculations.

We have checked the energetics of desorption and V_{Zn} diffusion processes using HSE functional. Because our slab supercells (containing 188 atoms with 25 Å vacuum gap and requiring a 4×2×1 k-points mesh) is too large for hybrid functional calculations, we used the relaxed structures in the LDA+U functional calculations. As summarized in Table S2, the LDA+U results are qualitatively similar to the HSE results.

Supplementary Table 2. Comparison of the LDA+U and HSE functional calculations.

	Desorption		Zn diffusion (layer)				
	Zn	O	0	1	2	3	4
LDA+U	0.3 eV	5.1 eV	0.0 eV	-0.2 eV	0.0 eV	0.4 eV	0.7 eV
HSE	0.4 eV	4.8 eV	0.0 eV	-0.3 eV	0.1 eV	0.5 eV	0.9 eV

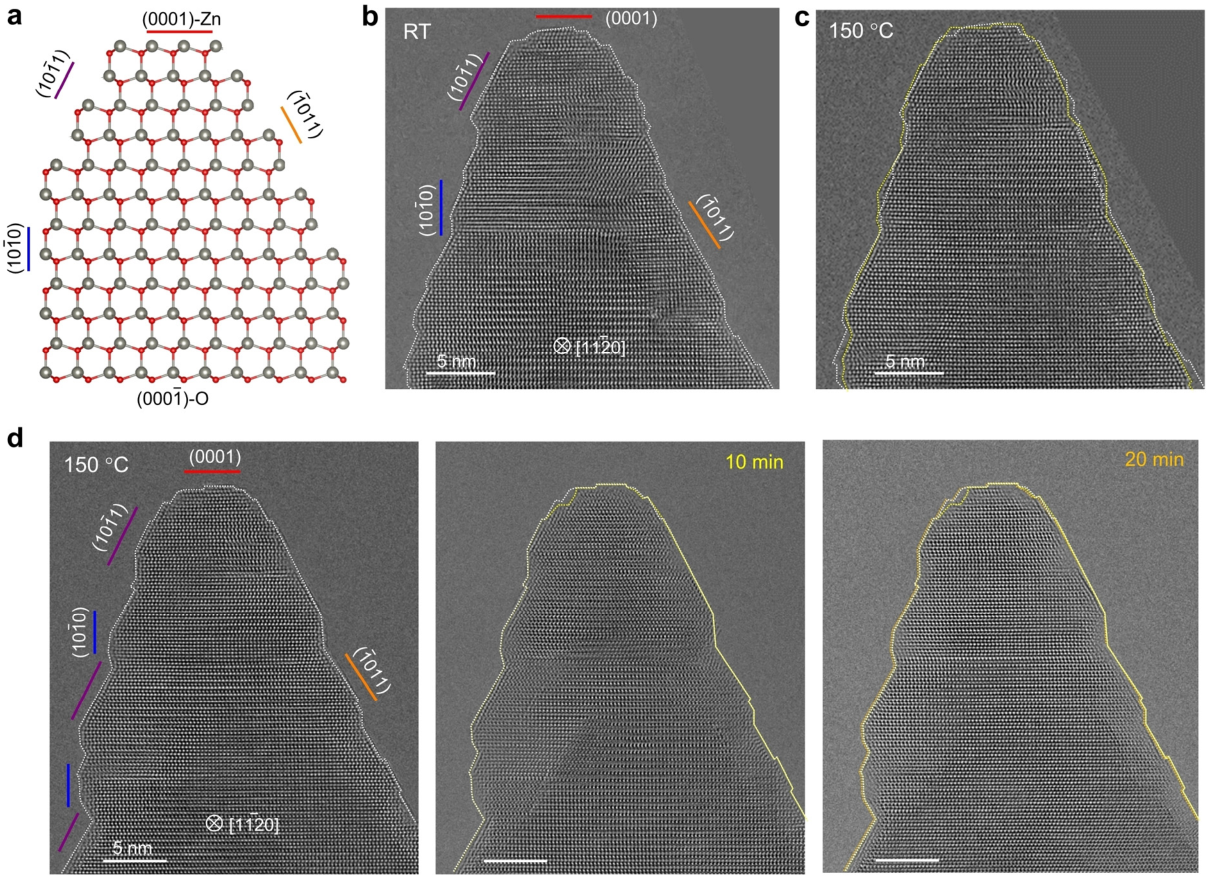

Supplementary Figure 1. A typical TEM sample of ZnO and low temperature evaporation behavior observed by HRTEM at 150 °C. a, Atomic model depicting the crystallographic orientation of one of the nanoscale protrusions of ZnO crystal formed by FIB milling. **b**, **c**, HRTEM image of the protrusion recorded at room temperature (RT) and 150 °C, respectively. The low index orientations such as polar (0001), semi-polar (10$\bar{1}$1), ($\bar{1}$011) and non-polar (10$\bar{1}$0) are indicated on the HRTEM images to guide the edge orientations of the protrusion. The zone axis is the [11$\bar{2}$0]. **d**, Time-series HRTEM images tracing the change of the ZnO protrusion at 150 °C. No significant change in the edge profile of the ZnO protrusion indicative of evaporation was observed after prolonged observation for 20 min at 150 °C except for slight changes due to knock-on displacement of surface atoms. The real-time HRTEM movie is provided as Supplementary Movie 1.

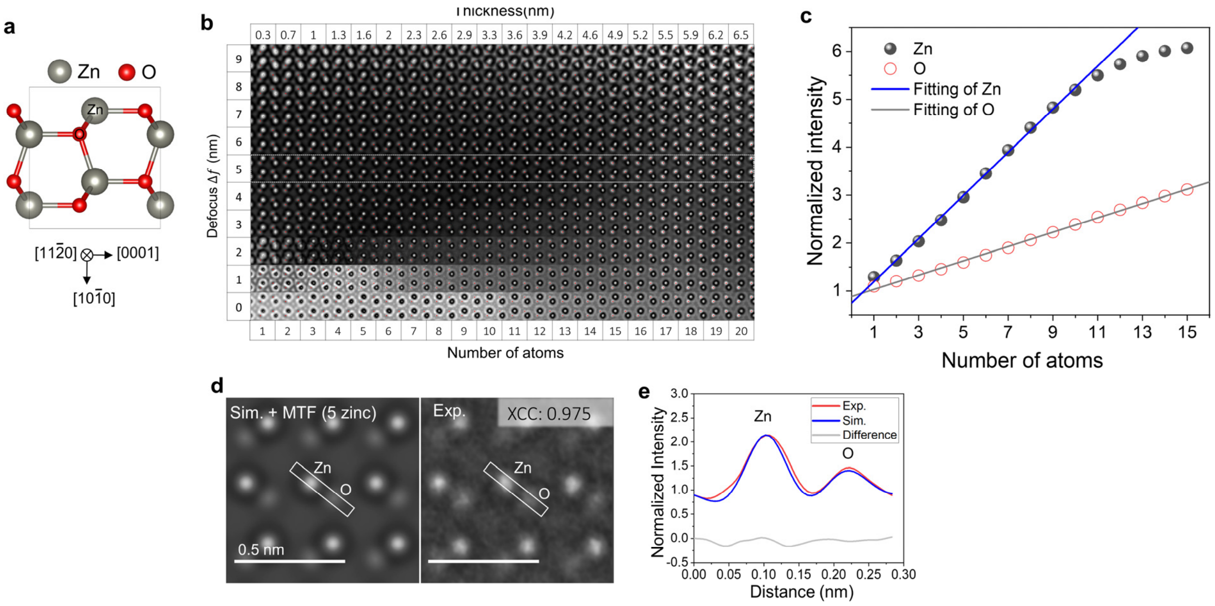

Supplementary Figure 2. Image simulation based quantitative analysis of in-situ NCSI HRTEM image for determination of the number of atoms in each column. a, Atomic model of ZnO unit cell used for HRTEM image simulation. The Zn and O atomic columns in the unit cell contains only one atom along the viewing direction. **b**, Thickness-defocus map of simulated HRTEM images. Thickness varies in the range of 1-20 unit cells in 1 unit cell step and defocus varies from 0 to 9 nm in 1 nm step. **c**, Variation of the normalized intensity of Zn and O column as a function of the number of atom in the corresponding column. For Zn, the linear relationship between the HRTEM intensity and the number of atom is maintained up to 10 atoms, equivalently 10 unit cells. For O, the linear relationship is valid up to 25 atoms. Within the validity of the linear relationship, the measured intensity can be used to determine the number of atoms in the column. **d**, Comparison of simulated (Sim.) and experimental (Exp.) NCSI HRTEM images. For the HRTEM image simulation, the modulation transfer function (MTF) function of complementary metal oxide semiconductor (CMOS) camera (Gatan^{TM} OneView) was included[3,4]. The simulated image for 5 unit cells in thickness and the defocus of +5.2 nm yields the highest cross-correlation coefficient (XCC) of 0.975 with the experimental HRTEM image which was cropped from the bulk region in Fig. 4a. **e**, Intensity profile of Zn and O atomic columns from simulated and experimental HRTEM images (white box in d). The two profiles match perfectly well with each other.

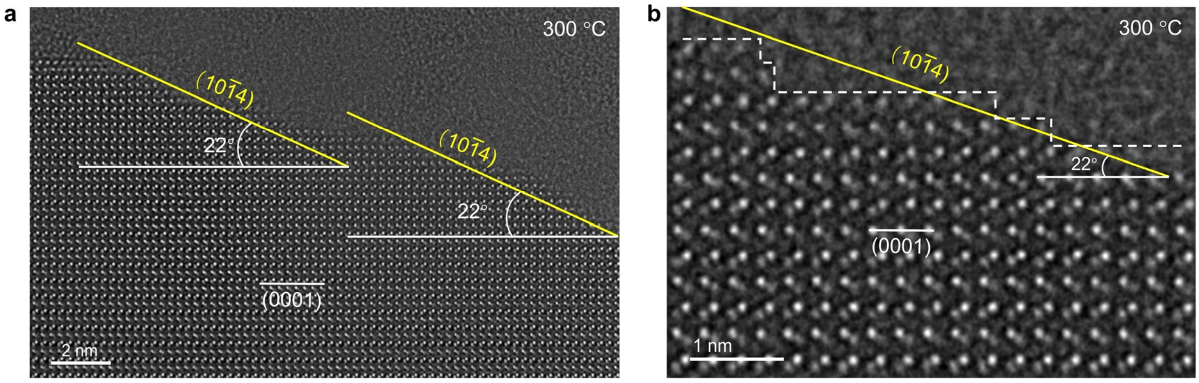

Supplementary Figure 3. Tendency of forming a vicinal orientation of the (0001) polar surface during evaporation. a, In-situ HRTEM image showing the orientation of moving SV interface of ZnO during evaporation at 300 °C. During the fast evaporation along the (0001) surface the moving SV interface often develops atomic steps which are regularly arrayed to form a vicinal surface orientation parallel to the $(10\bar{1}4)$ plane. **b**, Magnified view of the SV interface. The atomic steps and the $(10\bar{1}4)$ plane are outlined by white dash line and yellow line, respectively. The angle between the SV interface and the (0001) plane is measured to ~22°, which is close to the interplanar angle between the (0001) and the $(10\bar{1}4)$ planes.

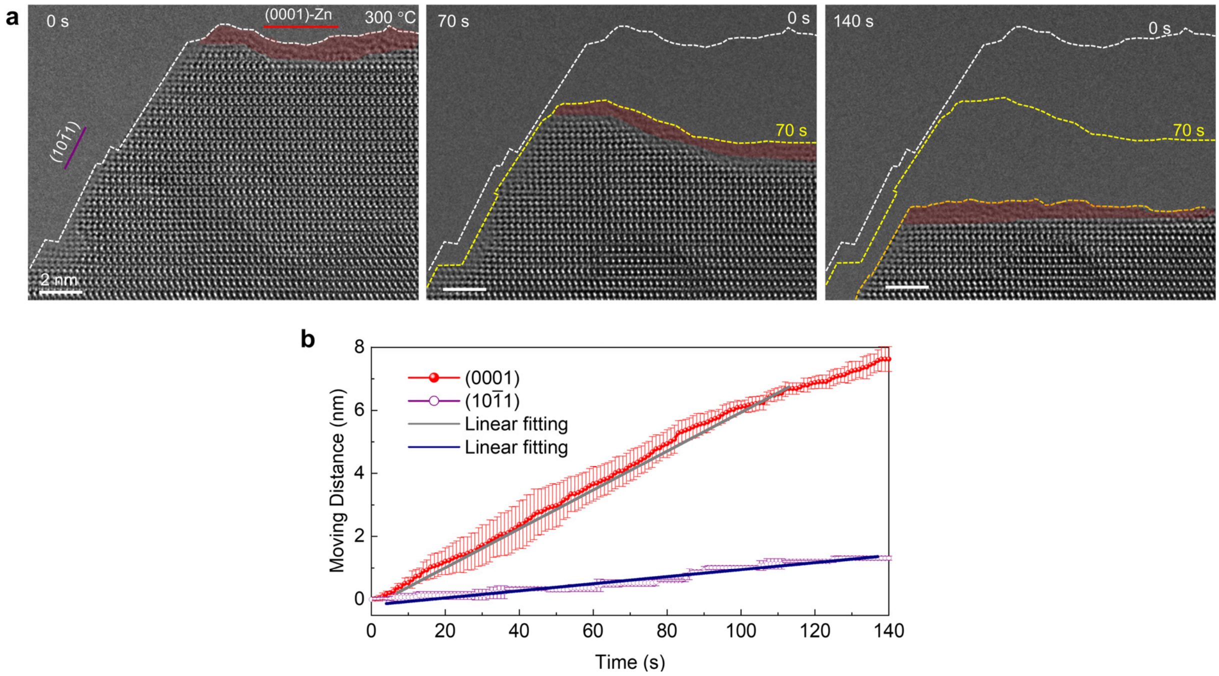

Supplementary Figure 4. Anisotropic evaporation behavior of the polar (0001) surface observed by in-situ HRTEM at 300 °C. a, Time-series HRTEM images of ZnO showing the formation of quasi-liquid layer (highlighted in red) on the (0001) surface at 300°C. **b**, Plot of the moving distance of the (0001) and the (10$\bar{1}$1) surface during evaporation. The moving rate from linear fitting of the (0001) surface was 0.06 nm s^{-1}, around 6 times higher than that of the (10$\bar{1}$1) surface (0.01 nm s^{-1}), which is similar to that shown in Fig. 2b. The error bars represent the standard deviation of several independent measurements at different region along [0001] direction. A sample thickness effect on the moving rate of each orientation is negligible as the thickness remains constant within the field of view.

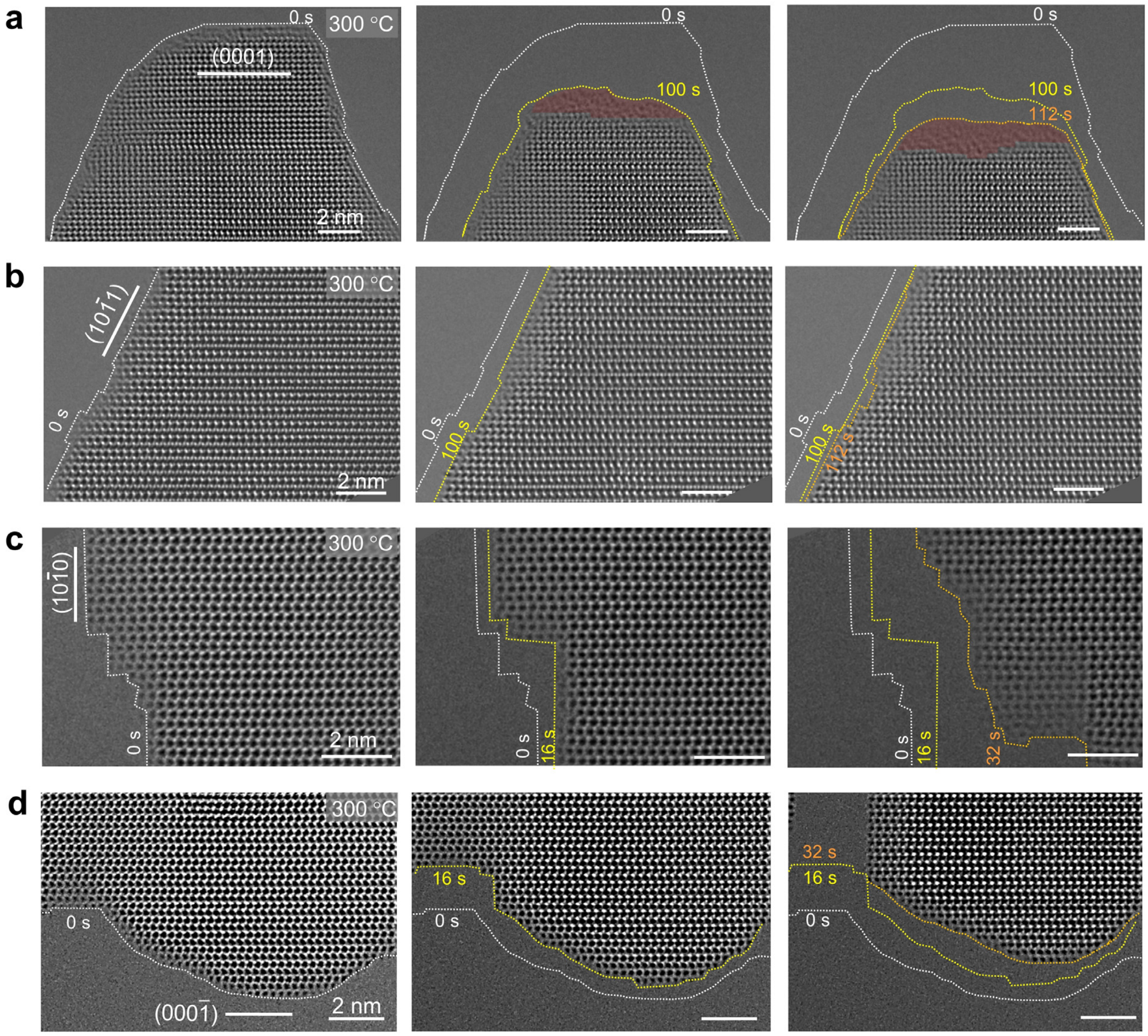

Supplementary Figure 5. Anisotropic evaporation behavior of ZnO observed by in-situ HRTEM at 300 °C. Time series HRTEM images of: **a**, polar (0001) surface; **b**, semi-polar $(10\bar{1}1)$ surface; **c** non-polar $(10\bar{1}0)$ surface; **d**, polar $(000\bar{1})$ surface edge. For each set of HRTEM images the surface edge was outlined in different color to trace its movement during evaporation. A quasi-liquid layer (highlighted in red) was formed only on the (0001) surface. The real-time HRTEM movies are provided as Supplementary Movie 2 and 4.

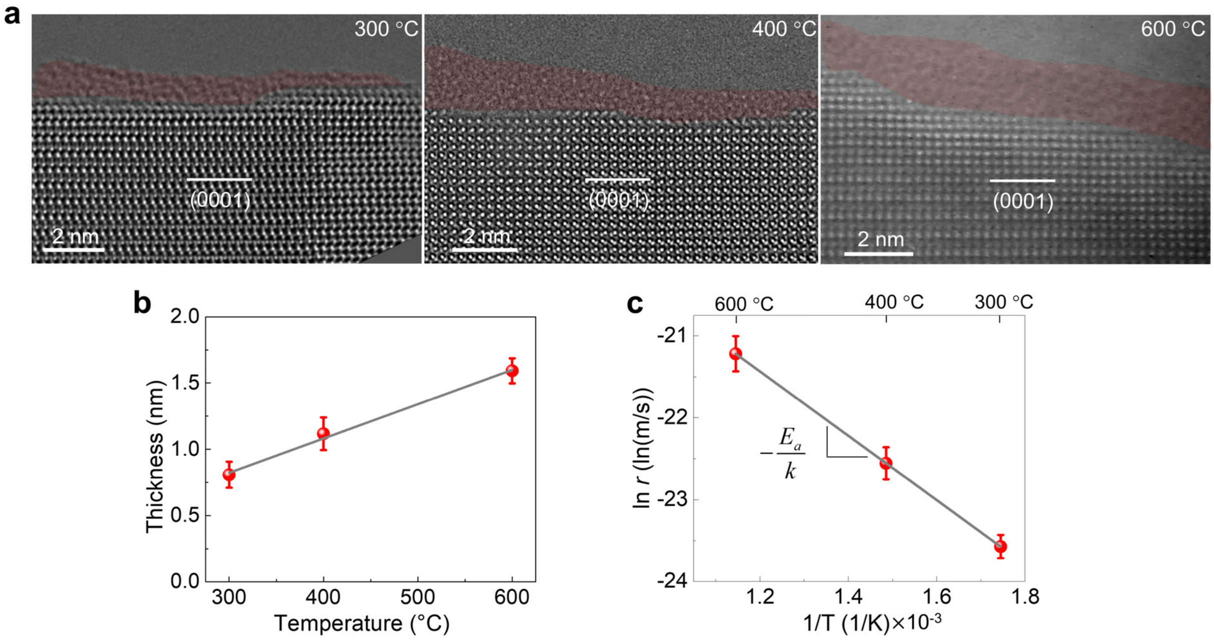

Supplementary Figure 6. Temperature-dependent growth of quasi-liquid layer and Arrhenius plot for the measurement of activation energy. a, In-situ HRTEM images showing the quasi-liquid layer (highlighted in red) formed on top of the (0001) polar surface during the evaporation at 300°C, 400°C, and 600°C. **b**, Plot of the thickness of quasi-liquid layer measured at different temperatures and a linear fit to the data. The error bars represent the standard deviation of several independent measurements of thickness at different region for each temperature. **c**, Arrhenius plot of the moving rate (*r*) of quasi-liquid layer. The activation energy determined from the slope of the linear fit is 0.34 ± 0.03 eV. The error bars represent the standard deviation of fittings of several independent surface moving rate measurements at different region for each temperature. We note that the electron beam irradiation stimulates but is not the major cause of surface disorder-mediated evaporation; the control experiments showed that the surface disordering indeed occurs on the (0001) surface under a reduced electron dose rate of ~10^3 e^- $Å^{-2}$ s^{-1} (Supplementary Fig. S10) or even in the absence of electron beam (Supplementary Fig. S9a).

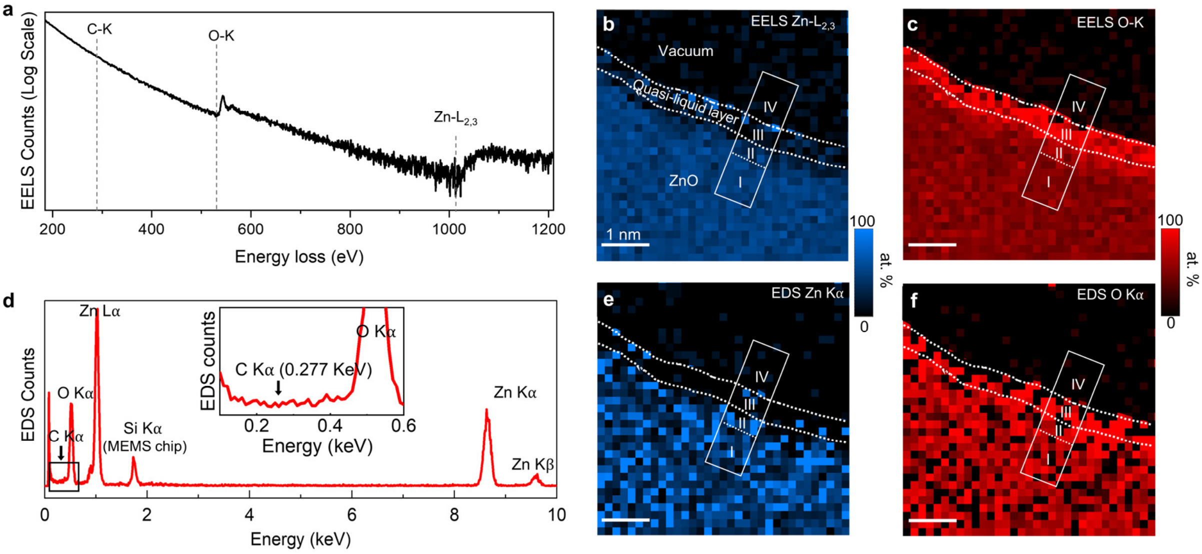

Supplementary Figure 7. In-situ STEM dual EELS and EDS elemental mapping. a, EEL spectrum showing Zn-$L_{2,3}$ and O-K edges used for elemental mapping and quantification. The absence of C-K edge indicates that there are no carbon based impurities in the sample. **b**, **c**, EELS atomic concentration (at. %) map of Zn and O, respectively. The region I, II, III and IV are defined by following the same convention as in Figs. 3a, b. The region II (subsurface crystalline $Zn_{1-x}O$) and III (quasi-liquid layer) is deficient of Zn compared to the region I (bulk crystalline ZnO). **d**, EDS spectrum showing characteristic Zn-K_{α} and O-K_{α} X-ray peaks used for elemental mapping and quantification. The EDS spectrum image has been acquired simultaneously with EEL spectrum image. No impurity such as carbon (inset) was detected except for Zn and O. Note that the Si-K_{α} peak originates from the MEMS chip where ZnO TEM sample is mounted. **e**, **f**, EDS atomic concentration (at. %) map of Zn and O obtained by selecting Zn-K_{α} and O-K_{α} peaks, respectively. Consistent with the EELS composition maps, the EDS composition maps also show Zn deficiency in the region II and III. The vacuum area with noise signal in **b, c, e,** and **f** are shaded.

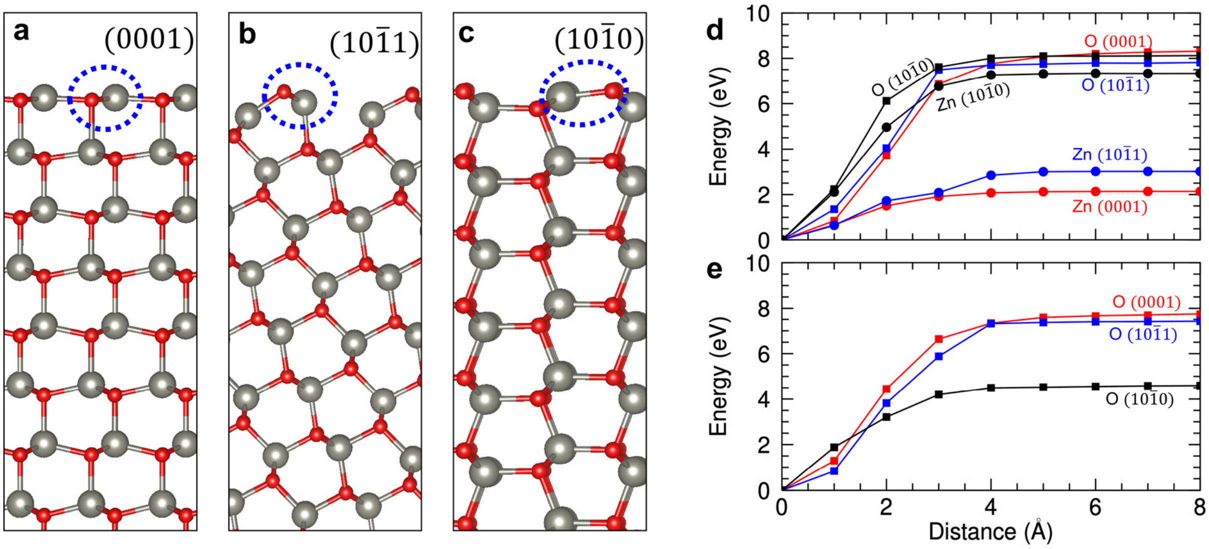

Supplementary Figure 8. Desorption energy barriers on various ZnO surfaces. a, **b**, **c**, Atomic structures of the ZnO (0001), (10$\bar{1}$1), and (10$\bar{1}$0) surfaces, respectively. A pair of surface Zn and O atoms considered for the Schottky defect formation is denoted using blue dotted circles. **d**, Energy variations in the Zn or O desorption, and **e**, Energy variations in the O desorption after the Zn atom in pair has been desorbed. The energy landscapes in **d** and **e** are represented as a function of the Zn or O distance from the corresponding surface. Because there is no uphill in the energy landscapes, the desorption barrier is determined from the plateau far from the surface. In other words, the desorption barrier of an atom X $E_{DB}(X)$ was calculated by $E_{DB}(X) = E(S\!:V_X + X^{away}) - E(S)$, where $E(S)$ and $E(S\!:V_X + X^{away})$ are the total energies of the pure surface and the surface containing a X vacancy (V_X) with a X atom far away from the surface, respectively. Here, $E(S\!:V_X + X^{away})$ is approximately the sum of the total energies of the surface with V_X $E(S\!:V_X)$ and the isolated X atom $E(X)$, i.e., $E(S\!:V_X + X^{away}) \cong E(S\!:V_X) + E(X)$, because the X atom is far away from the surface and considered as an isolated one. In our calculations, the energy differences between the left- and right-hand sides are below or about 0.1 eV for all the cases. On the other hand, the desorption energy of the X atom $E_{DE}(X)$ can be calculated by $E_{DE}(X) = E(S\!:V_X) + E_{ref}(X) - E(S)$, where $E_{ref}(X)$ is the reference energy of the X atom, i.e., $E_{ref}(Zn)$ and $E_{ref}(O)$ are the single atom energies of the Zn metal and O_2 molecule, respectively. Thus, the difference $E_{DB}(X) - E_{DE}(X) \cong E(X) - E_{ref}(X) = \Delta E(X)$, which is the energy change of a Zn or O atom associated with the formation of a Zn metal or O_2 molecule. In short, the desorption energies $E_{DE}(X)$ are smaller than the corresponding desorption barriers $E_{DB}(X)$ by the energy change $\Delta E(X)$, i.e., $E_{DE}(X) \cong E_{DB}(X) - \Delta E(X)$. In our calculations, $\Delta E(\mathrm{Zn})$=1.8 eV and $\Delta E(\mathrm{O})$=3.3 eV. In **e**, for examples, the O desorption barrier for the (0001) surface is 7.8 eV, and the corresponding desorption energy is 4.5 eV (= 7.8 eV – 3.3 eV).

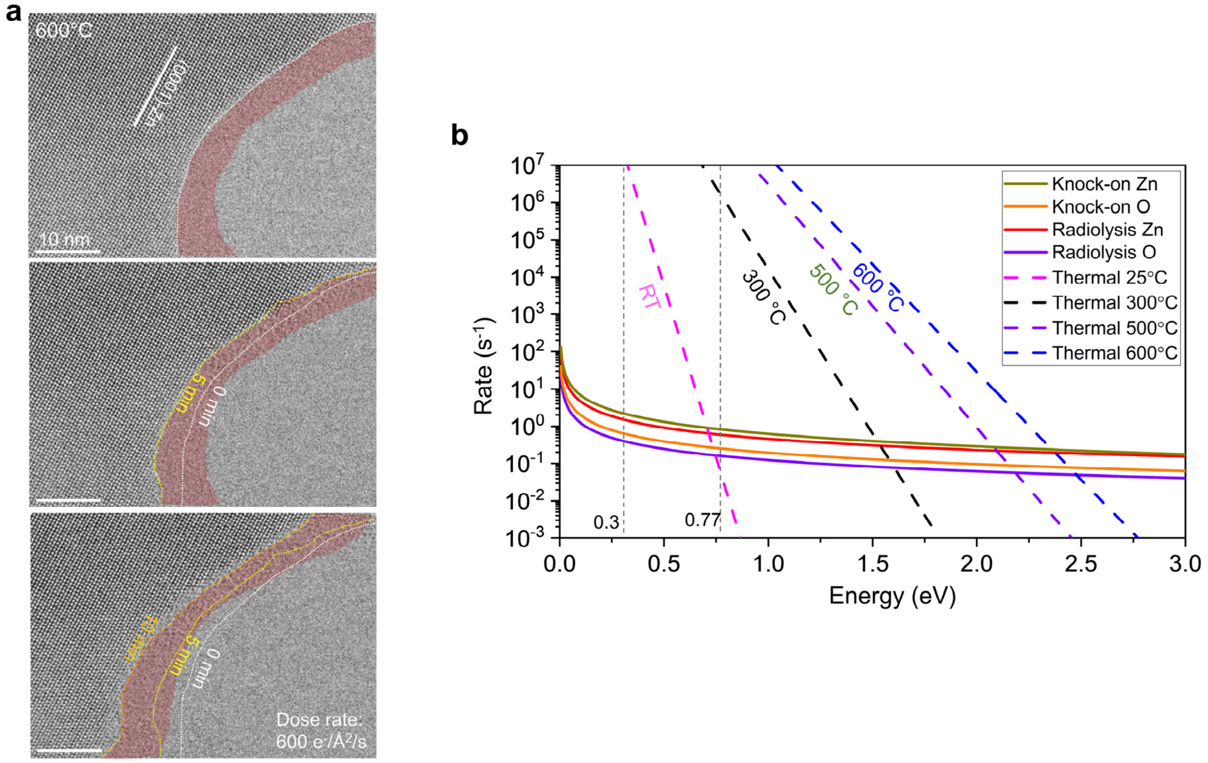

Supplementary Figure 9. Control experiment and theoretical calculation for evaluation of the electron beam effects on evaporation of ZnO. a, In-situ HRTEM images captured every 5 min. at 600 °C. To exclude the electron beam effects on the evaporation of ZnO, the electron beam was blocked throughout the heating experiment except for imaging. The formation of quasi-liquid layer and evaporation of ZnO occurred in the absence of electron beam at 600 °C. The crystal surface edge was outlined in different color dashed lines to trace its movement during evaporation. **b**, Comparison of the rate of electron beam-induced damage processes (radiolysis and knock-on displacement) with thermally activated processes (V_{Zn} formation and diffusion) with respect to the function of activation energy. The activation energy for the desorption of surface Zn atom (0.3 eV for our DFT calculation) and the inward diffusion of V_{Zn} (0.77 eV from our DFT calculation and the previous report by Erhart and Albe[8]) from the (0001) surface are indicated by vertical grid lines. Details on the calculation of the rates for electron beam damage processes are given in Supplementary Note 3.

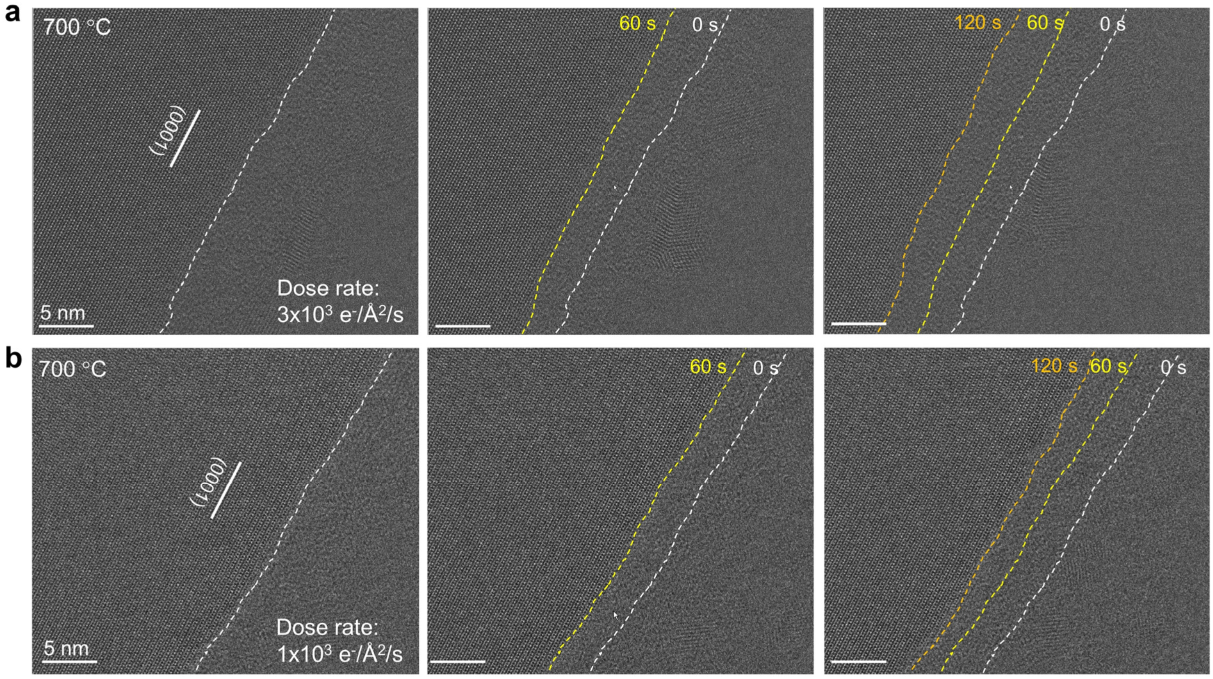

Supplementary Figure 10. In-situ HRTEM observation of ZnO evaporation under reduced electron dose rates. Time-series HRTEM images recorded at 700 °C under: **a**, ~3×10^3 e^- $Å^{-2}$ s^{-1}; **b**, ~1×10^3 e^- $Å^{-2}$ s^{-1}. The quasi-liquid layer was formed on the (0001) surface under such reduced electron dose rates. For each set of HRTEM images the surface edge was outlined in different color dash lines to trace its movement during evaporation. Note that most in-situ HRTEM experiments have been carried out under the electron dose rate of ~2×10^4 e^- $Å^{-2}$ s^{-1}.

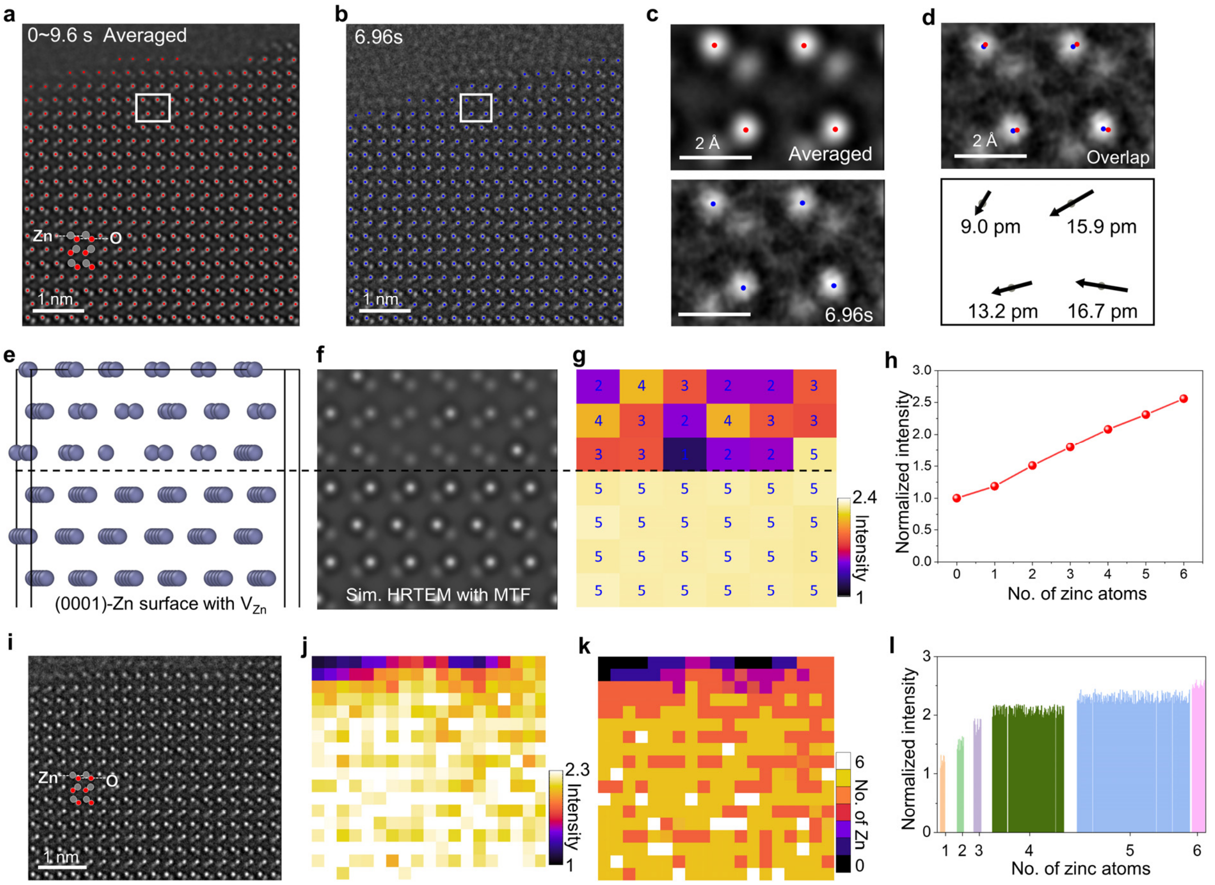

Supplementary Figure 11. Quantitative analysis of the position and intensity of Zn columns in in-situ NCSI HRTEM images for determination of displacement and V_{Zn}, respectively. a, 2D Gaussian fitted Zn positions overlayed on averaged HRTEM image from 0 to 9.6 s. **b**, 2D Gaussian fitted Zn positions overlayed on single shot HRTEM image at 6.96 s. **c**, Magnified images of white box area in **a** and **b**, showing the 2D Gaussian fitted Zn positions overlayed on averaged and single shot HRTEM images. **d**, Upper: Overlapped 2D Gaussian fitted Zn positions from averaged (red dots) and single shot (blue dots) HRTEM images; Lower: displacement map of Zn atomic columns from averaged positions to single shot positions. **e**, ZnO wurtzite structure with averaged 43% V_{Zn} on top three layers (above dash line), only Zn atoms are shown. 5 Zn atoms are contained for vacancy free region. Zn atom numbers in each column are shown as numbers in g. Zone axis is $[11\bar{2}0]$. **f,** Simulated HRTEM image of the structure in **e** convoluted with measured MTF function of OneView^{TM} (Gatan) CMOS camera. **g**, Intensity map from Zn columns, calculated from the simulated image of **f**. Color and number of each pixel represent the intensity and the number of Zn atom, respectively. The intensity in vacuum is normalized to 1. **h**, Normalized intensity plot as a function of number of Zn atoms from the simulated image. The intensity increases linearly with number of Zn atoms. A large range of linear relationship is shown in Supplementary Fig. S2c. **i**, A representative NCSI-HRTEM snapshot for extracting Zn column intensity. **j**, Extracted intensity map of each Zn column from HRTEM image in **i**. Intensity at vacuum is normalized to 1 for direct comparison with the intensity of simulated image. **k**, Calculated number (No.) of Zn atoms map from **j** by using the linear atom number-intensity relationship in **h**. **l**, Histogram of the normalized intensity from all Zn columns in **j**. The plot is arranged as a function of the number of Zn atoms.

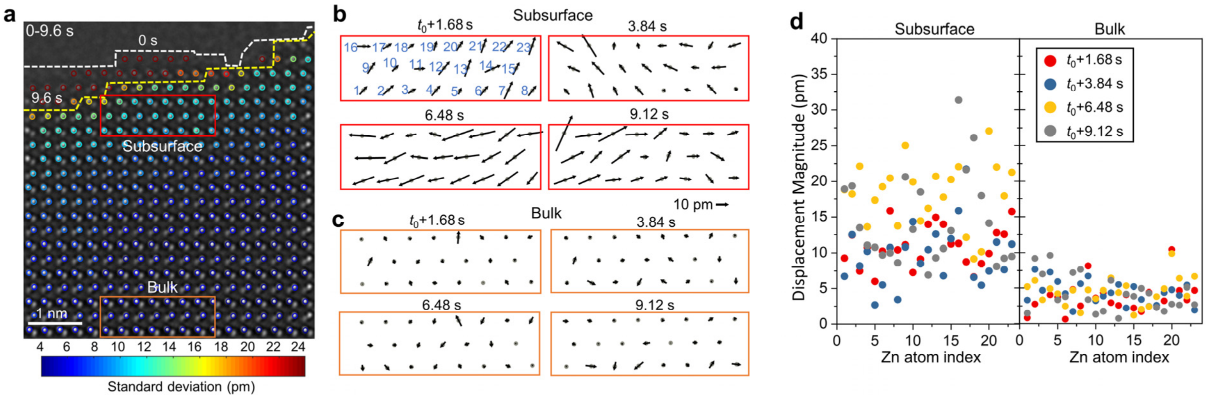

Supplementary Figure 12. High-precision displacement analysis of in-situ NCSI-HRTEM images. a, Displacement magnitude map of Zn atomic columns overlayed on HRTEM image. The displacement magnitude, displayed in colored circles, is represented by the standard deviation of averaged atomic column position for 9.6 s. The interface position of the quasi-liquid layer is indicated by dashed lines in different colors (white for 0 s and yellow for 9.6 s). While the displacement of Zn atoms in the bulk region is measured to be around 4~5 pm within the measurement precision, that of the subsurface Zn atoms reaches ~11-15 pm before disordering, which is about 10% of the Zn-O interatomic distance. **b**, **c**, Time-series displacement vector maps of the red and orange box regions in **a**, which represent the Zn-deficient $Zn_{1-x}O$ subsurface region and the bulk ZnO, respectively. **d**, Displacement magnitude of Zn atomic columns within the Zn-deficient subsurface region and the bulk region marked in **a**, respectively. The Zn atom indexes are marked in **b**, from 1 to 23.

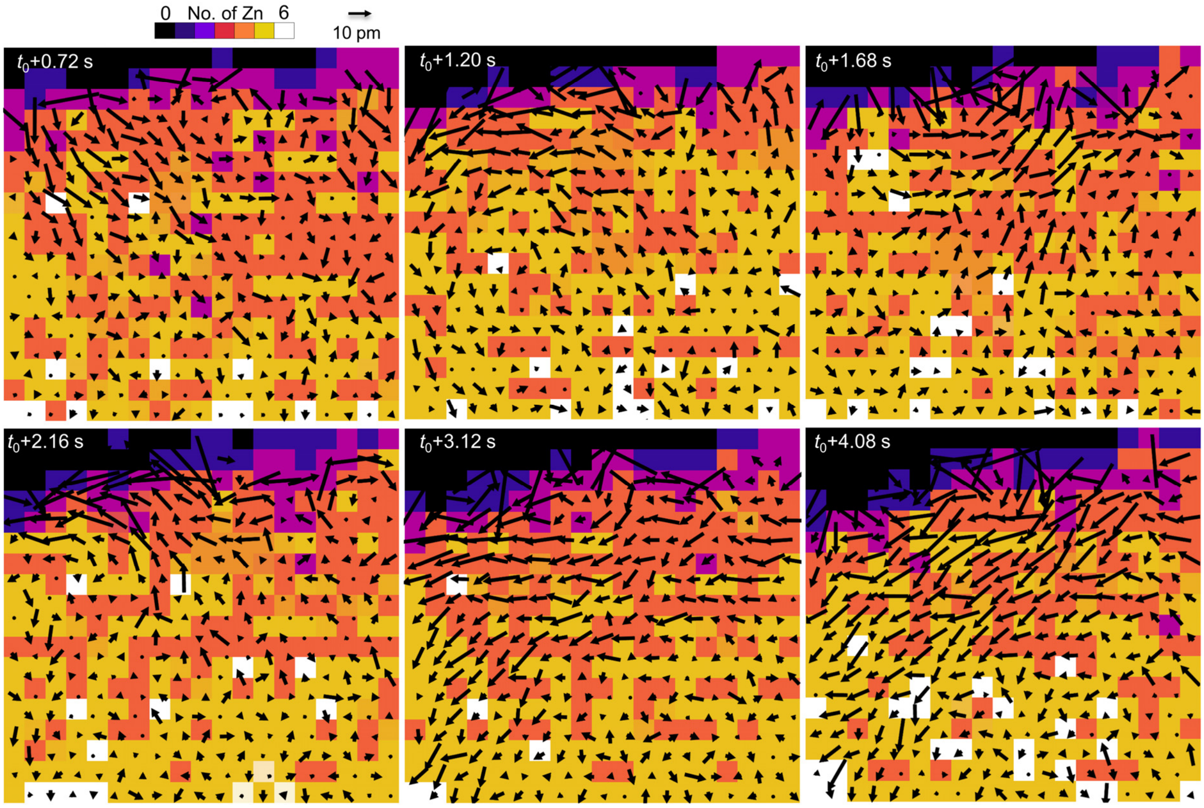

Supplementary Figure 13. Time series displacement vector (black arrow) and atom count maps (colored pixels) of Zn columns in the subsurface $Zn_{1-x}O$ region. The number of Zn atoms in each column was determined by quantitative analysis of the Zn column intensities. The displacement of each Zn and O atomic column was measured from 2D Gaussian fitted Zn positions of 0~9.6 s averaged HRTEM image shown in Supplementary Fig. S11. The magnitude of vector is multiplied by 30 times for better visualization. A high degree of correlation between V_{Zn} and displacement of Zn column is found; the Zn columns containing less Zn atoms, equivalently more V_{Zn}, exhibit large displacement in the subsurface region (Supplementary Movie 7).

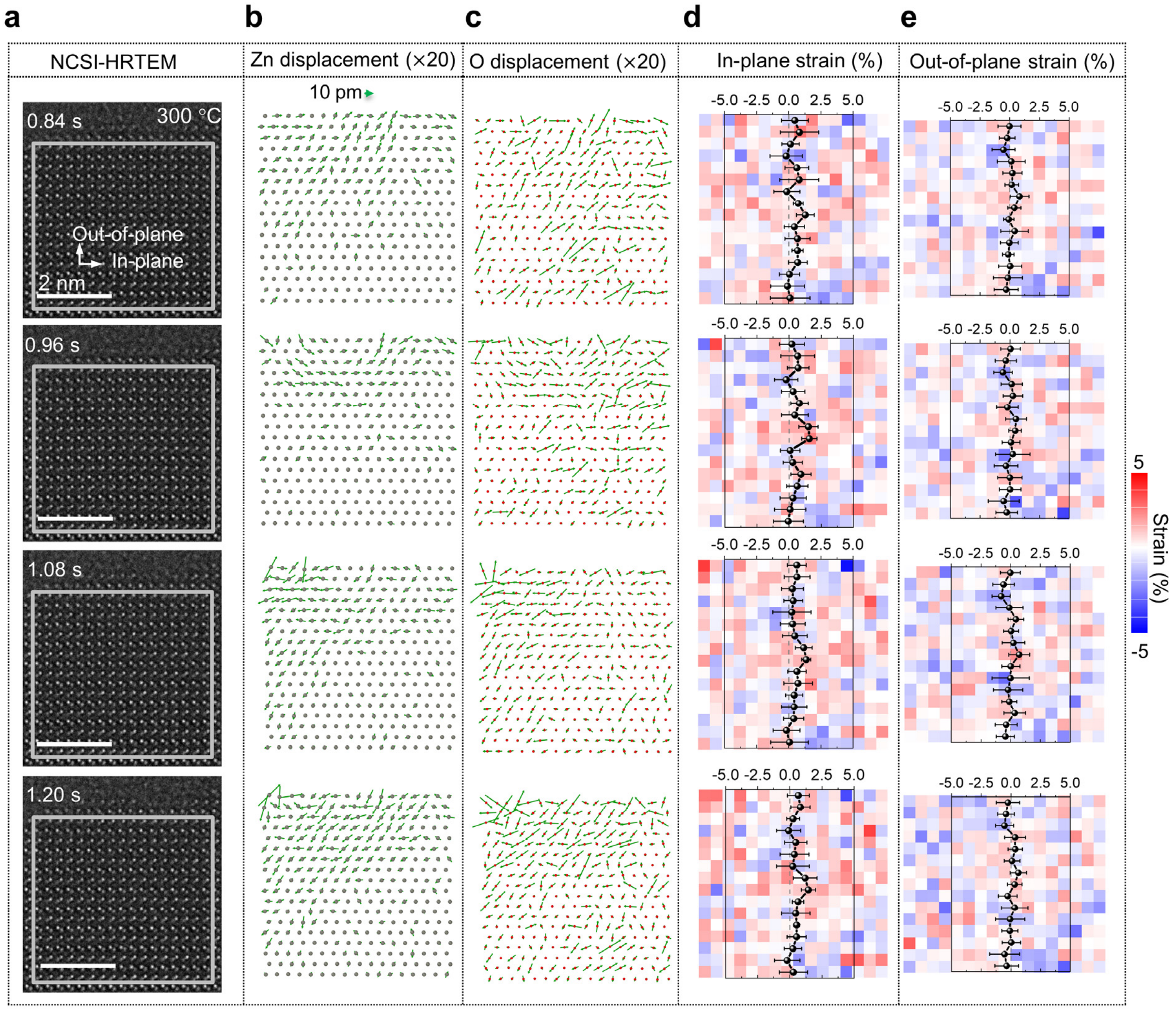

Supplementary Figure 14. Displacement and strain analysis of the atomic columns in subsurface $Zn_{1-x}O$ region. a, Time series NCSI-HRTEM images of Zn-deficient subsurface $Zn_{1-x}O$ under the quasi-liquid layer, where the diffusion of V_{Zn} results in the dynamic evolution of atomic displacement and lattice strain. **b**, **c**, Displacement maps of Zn and O columns within white box indicated in **a**, respectively. The displacement of each Zn and O atomic column was measured as the deviation from the average position over 9.6 s. The subsurface Zn and O columns show large displacements which are collectively coordinated each other. The magnitude of vector is multiplied by 20 times for better visualization. Note that the relatively larger displacements of O columns partially originate from the lower signal-to-noise ratio of O column intensity. **d**, **e**, In-plane strain and out-of-plane strain maps of the Zn sublattice. For each strain map the averaged strain profile is overlaid. The error bars represent the standard deviation of two strain components for each layer along in-plane direction in which they were averaged. Although the displacements of Zn and O result in local strain of the corresponding unit cell, they do not induce global external lattice strain (Supplementary Movie 8).

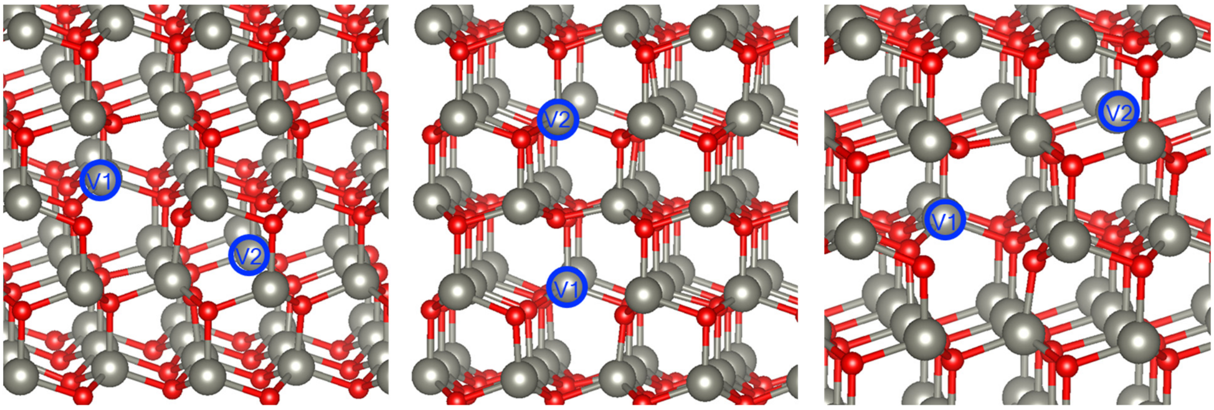

Supplementary Figure 15. Simulation cells for the elastic constant calculations. As one example, we show the three different supercells for 4% Zn vacancy concentration (two Zn vacancies in the cell). The positions of the Zn vacancies are denoted by blue circles, denoted by V1 and V2.

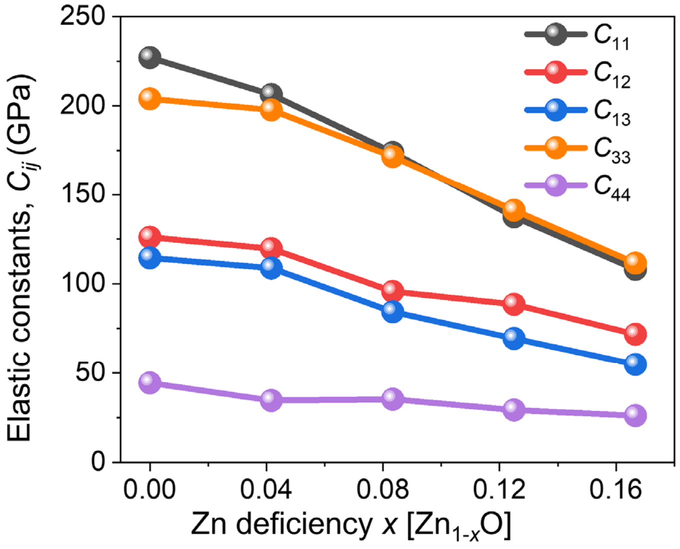

Supplementary Figure 16. Calculated elastic constants with respect to Zn deficiency x in $Zn_{1-x}O$. All the constants decrease almost linearly with the Zn deficiency x [$Zn_{1-x}O$].

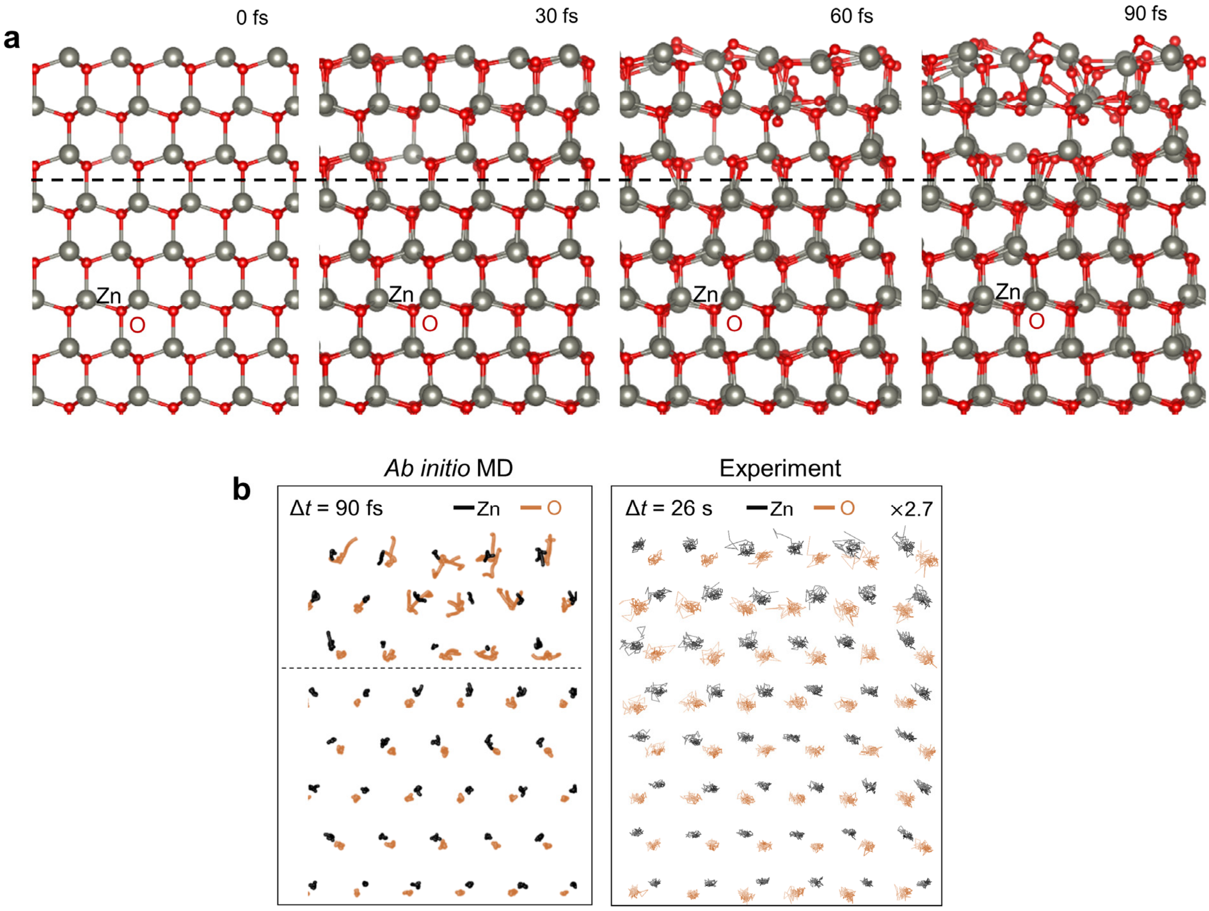

Supplementary Figure 17. ***Ab initio*** **MD simulation showing V_{Zn} induced disordering of ZnO.** **a**, Snapshots of MD simulation showing the disordering of top three Zn-O bilayers containing V_{Zn} (~43%) (Supplementary Movie 9). The dashed line marks the Zn-deficient layers on stoichiometric ZnO. **b**, Trajectories of Zn and O atoms in the *Ab initio* MD simulation traced for 90 fs (left) and those measured by using in-situ NCSI-HRTEM (Experiment) frame images (right). The displacement magnitude of O atoms in the Zn-deficient region is larger than that of Zn atoms.

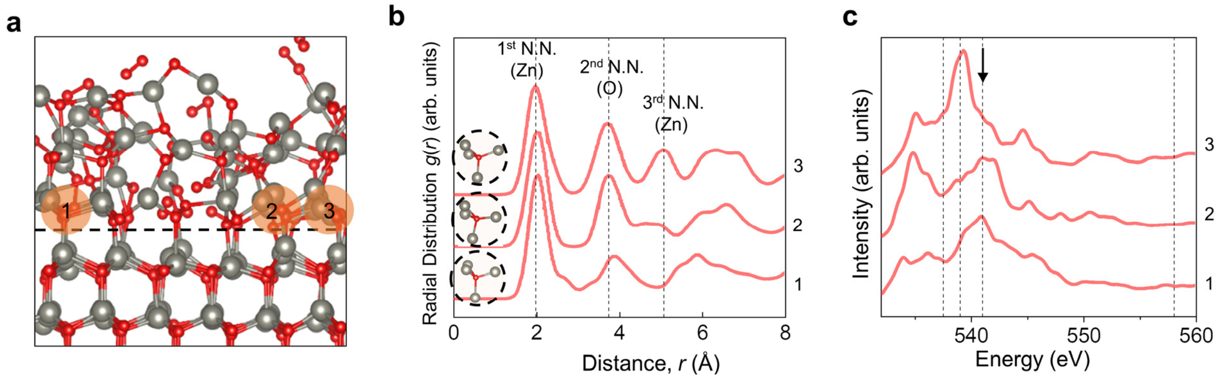

Supplementary Figure 18. Local atomic and electronic structure around V_{Zn}. a, Snapshot of *ab initio* MD simulation result at 0.45 ps. The dashed line marks the Zn-deficient layers on stoichiometric ZnO. The three quasi-liquid ZnO_4 tetrahedra with different atomic configuration are highlighted by orange circles and denoted by 1, 2 and 3. **b**, Radial distribution function (RDF), *g*(*r*) of quasi-liquid ZnO_4 tetrahedron denoted by 1, 2 and 3. The ZnO_4 tetrahedron 1 and 2 show the lack of 3rd nearest neighbor (N.N.) Zn. **c**, Calculated unoccupied oxygen DOS from quasi-liquid ZnO_4 tetrahedron denoted by 1, 2 and 3. The ZnO_4 tetrahedron 1 and 2 show an additional peak at around 544 eV (marked by black arrow), which does not exist in the ZnO_4 tetrahedron 3 with the 3rd N.N. Zn atom.

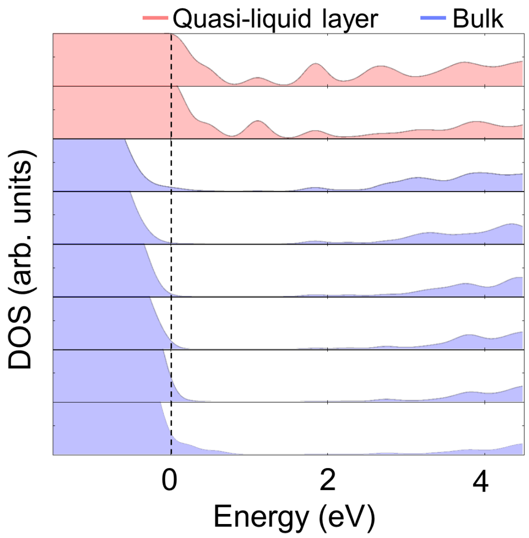

Supplementary Figure 19. Layer by layer DOS of ZnO slab with quasi-liquid layers from HSE calculation. The quasi-liquid layer (red area) still remains metallic even with HSE calculations, which is consistent with the standard DFT results shown in Fig. 6.

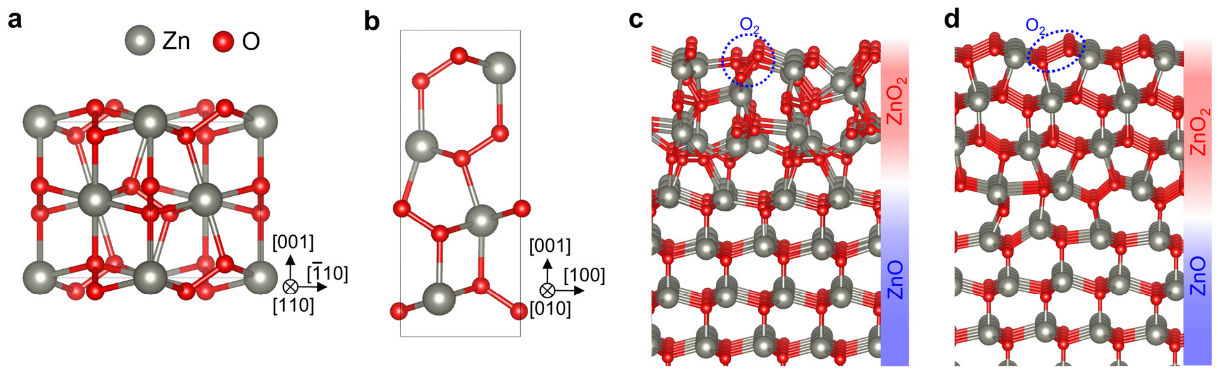

Supplementary Figure 20. Simulation cells constructed using ZnO_2 on ZnO to represent the evaporation of Zn-deficient $Zn_{1-x}O$ layer on ZnO. a, **b**, Unit cell of the two stable ZnO_2 structures, which have cubic and orthorhombic crystal structure, respectively. The viewing direction corresponds to the [110] and the [010] direction, respectively. **c**, **d**, Simulation cell constructed by placing ZnO_2 structure from **a** and **b** on top of (0001) ZnO surface, respectively. The thickness of ZnO_2 in **c** and **d** is 7.2 and 9.1 Å, respectively. The desorption energies of Zn, O and O_2 (indicated by dotted circles) calculated by DFT are plotted in Fig. 6e.

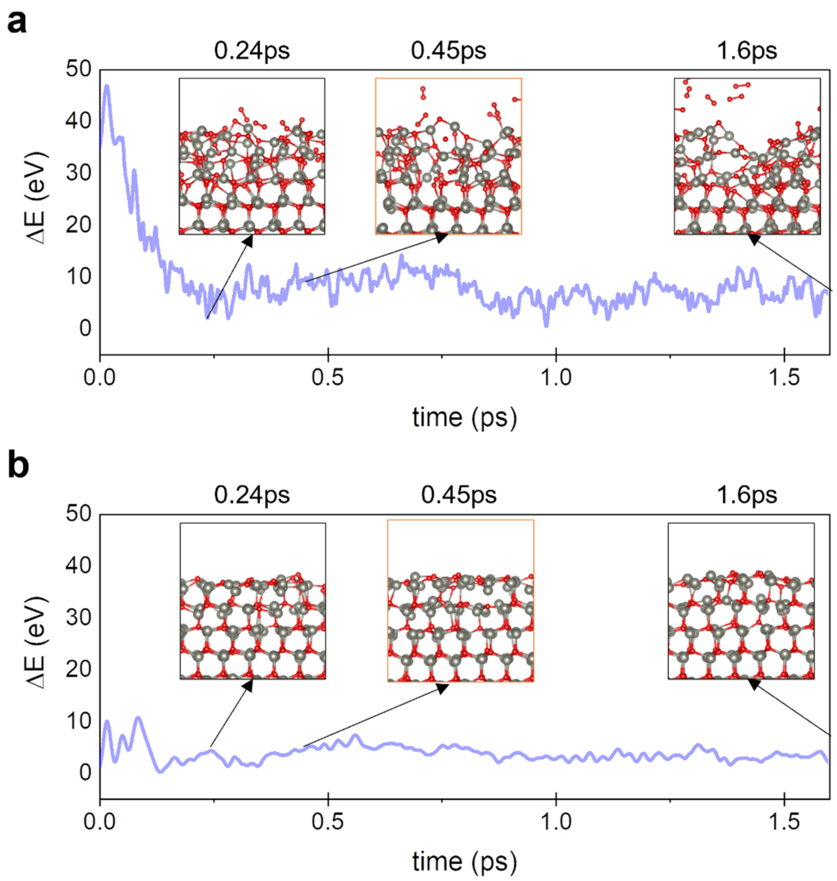

Supplementary Figure 21. Surface disorder of vacancy-stabilized ZnO polar surfaces. Relative energy of **a**, Zn-terminated (0001) surface with V_{Zn} and **b**, O-terminated $(000\bar{1})$ surface with oxygen vacancy as a function of time during MD simulation. Snapshots at 0.24, 0.45 and 1.6 ps are shown in the inset of figure. Quasi-liquid like disordered layer forms only on the (0001) surface.

Supplementary Movie 1. Low temperature evaporation of ZnO as observed by HRTEM at 150 °C. HRTEM movie of ZnO nanoscale protrusion recorded at 150 °C. No significant change in the edge profile of the ZnO protrusion was observed.

Supplementary Movie 2. Anisotropic evaporation of ZnO observed by in-situ HRTEM at 300 °C. At the early stage, the evaporation rate of the (0001) surface is measured to ~0.03 nm s^{-1} which is already higher than other surface orientations (~0.01 nm s^{-1}). The quasi-liquid layer starts forming at ~100 s only on the (0001) surface and the evaporation of the (0001) surface becomes accelerated steeply by more than 10 times. The surface disorder took place only on the (0001) polar surface, no similar quasi-liquid layer was observed from other surface orientations. Playing rate is 4 times accelerated for better visualization.

Supplementary Movie 3. NCSI HRTEM movie showing anisotropic evaporation of ZnO at 300 °C. NCSI HRTEM real-time movie (50 fps) was taken at the (0001) surface. Zn and O columns are clearly resolved under NCSI condition. The surface disorder took place only on the polar (0001) surface, no similar quasi-liquid layer was observed from other surface orientations such as $(10\bar{1}0)$ and $(10\bar{1}1)$.

Supplementary Movie 4. O-terminated and non-polar $(10\bar{1}0)$ ZnO evaporation at 300 °C. The polar $(000\bar{1})$ and the non-polar $(10\bar{1}0)$ surfaces evaporate rather slowly in a layer-by-layer manner without forming disordered quasi-liquid layer.

Supplementary Movie 5. NCSI HRTEM movie showing the atomistic details of disordering of (0001) surface at 300 °C. NCSI HRTEM real time movie (25 fps) was taken at the (0001) surface. Zn and O columns are clearly resolved under NCSI condition. A collective motion of surface zinc and oxygen atoms is noticeable before disordering.

Supplementary Movie 6. NCSI HRTEM movie showing the displacement of Zn and O atomic columns triggered by diffusion of Zn vacancies before the loss of long-range order. Zn and O columns are clearly resolved under NCSI HRTEM condition. A collective displacement of near-surface Zn and O atomic columns is noticeable before disordering.

Supplementary Movie 7. Collective motion from atomic displacement of Zn and O columns in Zn-deficient subsurface. Left panel is a NCSI HRTEM movie showing the dynamic motion of Zn and O atoms for 9.6 s with 40 frames. Each frame is prepared by averaging 6 consecutive frames for better SNR. Middle panel is the extracted 2-D Zn occupancy map from the Zn intensity. Right panel is the Zn and O atoms displacement map. The magnitude of displacement vectors is multiplied by 20 times for better visualization. Three panels are synchronized for direct comparison.

Supplementary Movie 8. Real-time strain mapping of NCSI HRTEM movie of ZnO during evaporation. Left panel is a NCSI HRTEM movie, which is the same one as in Supplementary Movie 7. Middle and right panels are the corresponding in-plane and out-of-plane strain calculated from the fitted Zn positions. No global external lattice strain is observed, although the displacements of Zn and O result in local strain of the corresponding unit cell. Three panels are synchronized for direct comparison.

Supplementary Movie 9. *Ab initio* MD simulations showing V_{Zn} induced lattice distortion and disordered quasi-liquid layer formation. Born–Oppenheimer MD simulations have been carried out in the microcanonical ensemble (NVE). Averaged 43% Zn atoms on top three layers were removed to generate V_{Zn}. As the simulation goes on, the (0001) polar surface with V_{Zn} becomes disordered. The O atoms show greater motion than the Zn. As the surface is getting more and more disordered, surface evaporation occurs with the formation of oxygen molecules (O_2), which desorbs from the top layer. The time span of the movie ranges from 0 to 0.45 ps.

Supplementary References

1. Jia, C. L. *et al.* Determination of the 3D shape of a nanoscale crystal with atomic resolution from a single image. *Nat. Mater.* **13**, 1044–1049 (2014).

2. Kauffmann, Y. HREM-DIMA. https://mtyaron.com/microscopy/hrem-dima/ (2013).

3. Thust, A. High-resolution transmission electron microscopy on an absolute contrast scale. *Phys. Rev. Lett.* **102**, 220801 (2009).

4. Van Den Broek, W., Van Aert, S. & Van Dyck, D. Fully automated measurement of the modulation transfer function of charge-coupled devices above the nyquist frequency. *Microsc. Microanal.* **18**, 336–342 (2012).

5. Nasrazadani, S. & Hassani, S. Chapter 2 - Modern analytical techniques in failure analysis of aerospace, chemical, and oil and gas industries. in *Handbook of Materials Failure Analysis with Case Studies from the Oil and Gas Industry* (eds. Makhlouf, A. S. H. & Aliofkhazraei, M.) 39–54 (Butterworth-Heinemann, 2016). doi:https://doi.org/10.1016/B978-0-08-100117-2.00010-8.

6. Egerton, R. F., Li, P. & Malac, M. Radiation damage in the TEM and SEM. *Micron* **35**, 399–409 (2004).

7. Lawrence, E. L., Levin, B. D. A., Boland, T., Chang, S. L. Y. & Crozier, P. A. Atomic Scale Characterization of Fluxional Cation Behavior on Nanoparticle Surfaces: Probing Oxygen Vacancy Creation/Annihilation at Surface Sites. *ACS Nano* **15**, 2624–2634 (2021).

8. Erhart, P. & Albe, K. Diffusion of zinc vacancies and interstitials in zinc oxide. *Appl. Phys. Lett.* **88**, 201918 (2006).
